# Systemic hypertension in adults with congenital heart diseases^☆^

**DOI:** 10.1016/j.ijcchd.2023.100456

**Published:** 2023-04-06

**Authors:** Jolanda Sabatino, Martina Avesani, Domenico Sirico, Elena Reffo, Biagio Castaldi, PierPaolo Bassareo, Giovanni Di Salvo

**Affiliations:** a Department of Women's and Children's Health, University of Padua, Padua, Italy; b Paediatric Research Institute (IRP), Città Della Speranza, 35127, Padua, Italy; c Unit of Adult Congenital Heart Disease, University College of Dublin, Dublin, Ireland

**Keywords:** Systemic hypertension-congenital heart disease-adults

## Abstract

Long-term effects of systemic hypertension (HTN) and HTN-mediated damages have been largely studied in non-congenital adult populations. By contrast, robust data about the predisposing factors, prevalence, consequences, and treatment of HTN in adults with congenital heart diseases (ACHD) is still scarce. Different mechanisms including the underlying cardiac disease, cardiac surgery and its consequences, the development of metabolic syndrome and secondary forms seem to play a role in HTN in ACHDs. To mitigate the potential long-term effects of HTN in this complex population, a meticulous follow-up is mandatory to identify patients who should receive treatment, and tailored strategies should be applied to obtain the best as possible result.

Thus, this review will investigate risk factors, effects, and treatments of HTN in ACHD patients.

## 1 Introduction

Long-term effects of systemic hypertension (HTN) and HTN-mediated damages have been largely studied in non-congenital adult populations. By contrast, robust data about the predisposing factors, prevalence, consequences, and treatment of HTN in adults with congenital heart diseases (ACHD) is still scarce.

Cut-offs for HTN are those at which the treatment advantages, secured either with lifestyle modifications or drugs, unquestionably overcome the treatment risks, as reported by multiple randomized clinical trials.

Accordingly, systemic HTN in ACHD is defined by the latest adult guidelines [1] as office SBP values of 140 mmHg or higher, and/or DBP values of 90 mmHg or higher. A European Consensus Panel on hypertension in children and adolescents [2] recently agreed, for 16-year-old adolescents or older, the values of ≥130/85 mmHg are adequate to diagnose systemic hypertension (HTN) in this specific population.

While long-term effects of HTN and HTN- mediated damage on the cardiovascular system, kidney, brain, and eye, have been largely studied in the non-congenital adult populations, robust data about the risk factors, prevalence, consequences, and treatment of HTN in adults with congenital heart diseases (ACHD) is still scarce.

Some explanations for this can be hypothesized. First, ACHDs and, even more, elderly ACHDs are a relatively recent growing epidemic. Secondly, clinicians may perceive life expectancy in patients with ACHDs as shorter and, as consequence, the prevention of acquired cardiovascular diseases as less important [3]. Lastly, the complexity of ACHD patients is high, and congenital aspects might be prioritized over preventive treatments in the busy clinical setting [4].

This review will present risk factors, effects, and treatments of HTN in ACHD patients.

## 2 Predisposing factors

Several mechanisms may be responsible for HTN in ACHD patients. Despite most of them presenting with “essential” hypertension, there are specific congenital heart diseases (CHDs) that need to be mentioned as having an intrinsic risk of HTN [5].

Coarctation of the aorta (COA) is the most well-known CHD at risk for HTN. Indeed, approximately 30% of children develop HTN after early repair of COA [6], and the prevalence increases up to 68% in adult studies, especially when investigated by 24-h ambulatory blood pressure monitoring [7,8]. Causes of HTN in COA are likely to be multifactorial. Increased aortic stiffness and reduced aortic distensibility, have been shown both in preoperative neonates with COA [9] and in patients after successful repair [10], notably in the pre-coarctation aorta and proximal arteries, suggesting intrinsic abnormalities in the aortic wall [11,12]. In addition, the decreased aortic distensibility was related to age at surgery implying that the longer the exposure to arch hypertension is, the higher the risk of irreversible arterial damage [13,14]. Abnormalities of smooth muscle, elastic fibers, and collagen in the ascending aorta have been confirmed in some histological analyses performed at the time of COA repair [15,16].

Aortic arch morphologies also seem to have an impact; in fact, gothic arch geometries are more associated with higher carotid artery intima-media thickness, stiffness index, and HTN than other morphologies, probably because of altered fluid dynamics in the ascending aorta [17,18]. Similarly, some surgical techniques, such as the subclavian flap repair, using non-aortic tissue, could alter the aortic wall elasticity and predispose to late HTN [19]. Furthermore, post-surgical residual narrowing or re-COA can trigger an increase in BP, which can persist after successful percutaneous treatment [20].

An acute aortic arch geometry after arterial switch operation has been described, suggesting an increased risk of hypertension in young adults [21,22] This could be particularly of concern because of the possible coexistence with coronary artery abnormalities.

Lastly, alterations in neurohormonal mechanisms have been described. An activation and an upregulation of the renin–angiotensin–aldosterone system as a consequence of renal hypoperfusion was shown before and after surgery [23]. Modifications in baroreceptors functions before and after COA repair are also reported [24,25]. Indeed, the reduced arterial wall compliance, deriving from the morphological arterial wall abnormalities cited above and from chronic HTN, could depress the baroreflex, influencing baroreceptors to tolerate a higher pressure.

The role of HTN in COA is of particular concern because despite a successful repair survival is still lower than in the general population and accelerated atherosclerosis is the main cause of premature death [26]. Thus, a lower threshold toward traditional CV risk factors is recommended.

Abnormalities in great arterial medial wall components potentially increasing aortic stiffness have also been documented in 18 other CHDs, including Tetralogy of Fallot, common arterial trunk, single ventricle physiologies, and D-transposition of great arteries [15,27]. However, whether these abnormalities are inherited or acquired, is still unknown.

Supravalvular aortic stenosis and renal stenosis, typical features of William Syndrome also induce systemic hypertension [28,29], and impairment of aortic wall elasticity was recently found to be an early change in patients with bicuspid aortic valve (BAV), thus increasing aortic stiffness and potential risk of HTN [30].

Lastly, patients with Turner Syndrome, a genetic disorder with several cardiovascular abnormalities, often associated with COA and BAV, have an increased prevalence of HTN, up to 30%, particularly nocturnal [31,32].

Beyond the above-mentioned specific diseases, CHDs share some features that can predispose to chronic kidney damage and, consequently, to HTN. For example, in cyanotic CHDs chronic hypoxia induces the production of erythropoietin, thus increasing erythrocytosis and blood viscosity [33]. Hyperviscosity induces an increase in efferent arteriolar tone with resulting glomerular hypertension, which can provoke chronic nephropathological changes including glomerulosclerosis [34].

Cardiac surgery itself provokes a disturbance of cardiac receptors, causing an enhanced sympathetic activity [35] and potential development of postoperative acute [36,37] and chronic kidney damage [38], either clinical or subclinical and especially in patients with complex cardiac anatomies who require longer bypass time and who are more exposed to renal hypoperfusion [39]. Also, atrial and brain natriuretic peptides, renin, aldosterone, and norepinephrine, important regulators of renal physiology, were found to be persistently elevated in patients with different CHDs years after surgical correction [[40], [41], [42]]. All these conditions, as well as chronic volume overload and the use of drugs (i.e. diuretics and angiotensin-converting enzyme inhibitors), can induce glomerulosclerosis and an increase of mesangial matrix, resulting in long-term chronic kidney damage [43].

A sedentary lifestyle and physical inactivity are negative behaviors that increase cardiovascular risk in ACHDs and are associated with HTN [43]. Some studies demonstrated that obesity *per se* is associated with worse cardiac remodelling and function in patients with COA successfully corrected [7]. Although some patients with more complex CHDs might have real limitations in their ability to perform physical activities, most of them are more likely to face social barriers. Indeed, despite the importance of a physically active lifestyle is recognized by consensus statements [44], parental overprotection, medical restrictions, inadequate patient education and the shortage of dedicated cardiac rehabilitation programs are still major limiting factors in the promotion of a healthy lifestyle in ACHDs [[45], [46], [47]].

Lastly, physicians should remember that adult patients with CHDs are special, but not aliens. Thus, causes of secondary hypertension and the presence of acquired modifiable cardiovascular risk factors (i.e diabetes, smoking, obstructive sleep apnoea) should always be kept in mind and investigated in case of high suspicion or in young adult patients with BP values only partially explained by their clinical conditions [1].

The presence of renal disease, either parenchymal, atherosclerotic, or fibromuscular, can be assessed with blood and urinary tests, as well as renal ultrasound or computed tomography. Endocrine causes such as primary aldosteronism, phaeochromocytoma, Cushing's syndrome, thyroid, or parathyroid disorders have specific clinical symptoms and signs, and the final diagnosis is based on blood or urinary tests. Medications such as oral contraceptive pill, diet pills, steroids, non-steroidal anti-inflammatory drugs, as well as substances like liquorice and stimulant drugs may also increase BP or antagonize the BP-lowering effect of antihypertensive medications; as consequence, a careful drug history is important when considering a diagnosis of secondary hypertension. Lastly, some rare genetic disorders causing HTN in children, adolescents, or young adults exist. Most of them induce hypertension by increasing the renal tubular reabsorption of sodium and are characterized by a suppressed plasma renin concentration or plasma renin activity [1].

## 3 Effects of systemic hypertension

The long-term effects of HTN and HTN-mediated damage on the cardiovascular system, kidney, brain, and eye, have been largely studied in the non-congenital adult populations [1]. By contrast, robust data in adults with congenital heart disease (ACHD) is still scarce, since elderly ACHDs are a relatively recent growing epidemic.

The prevalence of HTN in ACHD patients varies in different studies between 21% and 47%, likely depending on the age of the population included [3,48,49]. Men seem to be more affected than women, and overall HTN is more common among patients with less severe CHDs than in those with severe forms, probably because the latter die before the development of this condition [50,51].

The effects of prolonged exposition to high BP in CHDs are understudied. A large study on 6933 patients (median age 32 years) found HTN unrelated to all-cause of cardiovascular mortality [52]. By contrast, more recent data from Jepson et al. identified HTN as one of the risk factors for adverse outcomes such as stroke, myocardial infarction, surgical intervention for aortic aneurysm, aortic dissection, atrial arrhythmias, cardiac transplantation and death in a study on 1070 ACHD patients of similar age [48]. The different population samples may explain these different results. The association between HTN and atrial arrhythmias has been largely studied, not only in the general population but also in ACHDs [53]. Similarly, the consequences of HNT on morphology and function of the heart are well-established; chronic pressure overload induces ventricular hypertrophy and fibrosis, diastolic and systolic dysfunction, atrial and aortic dilatation, abnormal activity of the cardiac sympathetic nervous system and an increased risk of arrhythmias [54]. Those features are of concern when associated with a heart already affected by congenital abnormalities and the surgical sequel.

Lastly, ACHD patients can have moderate residual lesions, such as re-coarctation, aortic stenosis, subaortic membrane, known or unrecognized. These can cause hypertrophy and act as confounders, making the differential diagnosis between HNT and congenital residual defects tricky, especially in the context of clinical appointments, where BP measurements may be altered by stress factors [1].

Considering all this, a meticulous and periodical evaluation of cardiovascular risk in patients with ACHDs is mandatory for the correct prevention and management of HNT. It is still controversial if based on these data the same cut-off values used for the general population should be applied for specific CHD associated with accelerated atherosclerosis.

## 4 Diagnosis and classification

**Fig. 1:**
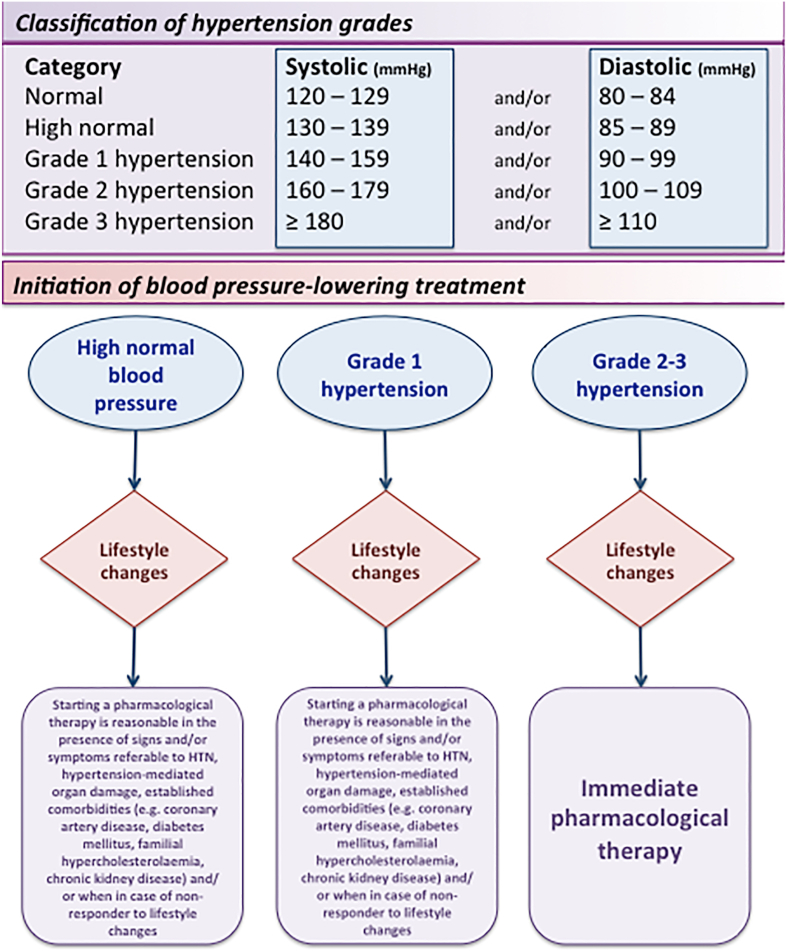
Classification of Hypertension Grades.

Classification of hypertension grades is reported in Fig. 1.

BP is commonly measured by office BP (OBP) recordings, Ambulatory Blood Pressure Monitoring (ABPM), and Home Blood Pressure Monitoring (HBPM).

OBP needs to be recorded after the ACHD patient has been sitting relaxed for 5 min, with the arm resting and supported at the heart level. According to the auscultatory method, systolic BP corresponds to the appearance of the first Korotkoff's tone, and diastolic BP to the complete disappearance of the tones (5th Korotkoff's) [1]. OBP should be measured at least three times and calculated by averaging the last two measurements, often discarding the first. In ACHD patients, BP should be compulsorily assessed in both arms and one leg, with the subject in the supine position, to rule out aortic coarctation [1]. To secure the diagnosis, HTN should be confirmed in a second outpatient assessment after a few weeks.

ABPM consists of multiple BP readings generally over a 24 h period [1]. The diagnostic threshold for ABPM hypertension over 24 h is 130/80 mmHg or higher (≥135/85 mmHg during the daytime; ≥120/70 during the night-time) [1].

During sleep BP is expected to decrease; ACHD patients can be defined as ‘dippers’ when their nocturnal BP diminishes by more than 10% of the daytime BP, and those with a paradoxical rise during the night reading are classified as reverse dippers. Various conditions are causing an absence of nocturnal BP dipping, that ACHD patients share with the general population, such as obesity, obstructive sleep apnoea, sleep disturbance, high salt intake, diabetic neuropathy, autonomic dysfunction, CKD, and old age [1]. A non-dipping pattern is associated with an increased risk of cardiovascular events, target organ damage, future cerebrovascular events, and secondary hypertension. Non-dippers are more likely to experience left ventricular hypertrophy (LVH), carotid intima-media thickening, microalbuminuria, and cerebrovascular diseases. Long-term clinical trials have demonstrated that administering medications during the night can improve dipping patterns and reduce the risk of cardiovascular events [55,56].

The advantages of ABPM are to reveal unreal high BP due to anxiety, such as white coat hypertension (WCH), and to assess circadian BP [1]. If OBP and ABPM are both normal, an ACHD patient can be considered normotensive. When the BP is elevated by OBP, but normal as measured by ABPM, most probably the ACHD patient has WCH; when the opposite is true, the patient has masked hypertension [1].

However, when examining ACHD with ABPM, one should take into consideration the scarce compliance of some adolescents or patients with genetic syndromes, especially during the night, which can false interpretations and measurements. Other disadvantages are, surely, the device cost and the limited availability, as well as its uncomfortableness.

HBPM consists of the average of all BP recordings utilizing a semiautomatic and validated BP monitor, for a minimum of 3 days to at least 6–7 consecutive days before an outpatient visit [1].

It is undoubtedly much cheaper and more available than ABPM. Another advantage is its clinical relevance to providing multiple measurements over longer periods. On the other hand, its major drawbacks are the possibility of measurement errors and the absence of nocturnal readings.

## 5 Treatment

### 5.1 Lifestyle modification

According to the newer recommendations, the management of HTN should start with non-pharmacological interventions [[41], [42], [43], [44], [57]]. Lifestyle modification is the primary action to be taken, to delay the drug treatment or to boost the BP lowering effect of pharmacological interventions.

General recommendations for lifestyle modification in hypertensive ACHD are summarized below.

- Avoid sedentary behavior
- Physical training should be encouraged (establishing realistic goals)
- Tailored diet with a graduate weight-loss program should be encouraged
- Avoid free sugar and saturated fat.
- Fruits and vegetables are encouraged
- Sodium intake should be restricted
- Reduce/avoid smoking

In general, even if regular physical exercise is related to a lower risk of obesity and/or future acquired cardiovascular disease in ACHD, physicians have been over-conservative in sports prescription in the past few decades. On the contrary, children and adults with congenital heart disease tend to be encouraged towards a sedentary lifestyle in virtue of general overprotection [[43], [44], [57]], and/or uncertainty concerning which sport and with what intensity could be safely practiced.

Even ACHD with symptoms should not be discouraged from performing physical activity. Physical exercise capacity should be tested by cardiopulmonary exercise testing (CPET) before prescribing sports in ACHD, to provide parameters, such as heart rate reserve, maximal heart rate (MHR), maximal/peak oxygen consumption (peak-VO2), allowing individualized physical activities.

In hypertensive ACHD, dynamic exercise is more advisable than static exercise, as dynamic exercise demands mainly a volume load, while a pressure load is principally produced by static exercise.

Overall, most ACHD patients can safely participate in regular and moderate physical activities. The use of a wearable device to monitor heart rate, ECG, SpO 2, should be encouraged and could be of help to encourage patients and physicians in promoting physical activity. A few diseases, such as pulmonary hypertension, systemic ventricular systolic dysfunction, malignant ventricular arrhythmias, systemic outflow tract obstruction, or significant aortic dilation, necessitate more vigilance.

### 5.2 Pharmacological treatment in hypertensive ACHD

Starting a pharmacological therapy is reasonable in the presence of signs and/or symptoms referable to HTN, hypertension-mediated organ damage, stage 2 HTN, established comorbidities, and/or when in case of non-responder to lifestyle changes [1] (Fig. 1). Five major drug classes are generally recommended in this specific setting: angiotensin-converting enzyme ACE inhibitors (ACEI), angiotensin receptor blockers (ARBs), beta-blockers, calcium channel blockers (CCBs), and diuretics (Table 1).

**Table 1:** Anti Hypertensive medical treatment in ACHD.

Drug Class	Advantages	Disadvantages
*Inhibitors of Renin–Angiotensin–Aldosterone System*	• Recommended in systemic LV failure. • ARBs may slow the progression of aortic dilatation in aortopathies. • Sacubitril/valsartan in patients who persist symptomatic regardless of optimal HF therapy.	• In Fontan patients the afterload reduction may have a detrimental effect on cardiac filling and output • May exacerbate a pre-existing right-to-left shunt • Should be discontinued during pregnancy
*Beta-blockers*	• Recommended in ACHD with heart failure (also systemic RV and HFpEF). • Labetalol can be used in pregnancy	• May exacerbate conduction abnormalities • Should be discontinued during pregnancy (detrimental effects on the conduction system in the fetus; birth defects)
*Diuretics*	• Recommended in decompensated heart failure with preserved or reduced ejection fraction, even in Eisenmenger syndrome with a right-to-left shunt. • Spironolactone improves the overall survival in ACHD patients with heart failure.	• Inappropriate dose increase of all diuretics can reduce preload in Fontan patients, and, in turn, cardiac output.
*Calcium antagonists*	• Non-dihydropyridines are used in hypertensive ACHD, who also require rate control for supraventricular arrhythmias. • Dihydropyridines are powerful vasodilators and need to be used when a more aggressive blood pressure control is needed. • Nifedipine is efficacious and safe in pregnancy-induced hypertension	• Non-dihydropyridines: important negative inotropic and chronotropic effects in ACHD with systemic left ventricular failure, • Non-dihydropyridines: may unmask latent conduction system disease (such as in ACHD with pre-excitation or Ebstein anomaly of the tricuspid valve) • Dihydropyridines: in ACHD with shunt defects may exaggerate a right-to-left shunt

Antihypertensive medications are often initiated as monotherapy after conservative management with lifestyle changes has failed. Recent guidelines recommend using thiazide-type diuretics or CCBs as first-line therapy, alone or in combination with other antihypertensive medications, for all patients with hypertension, except for those with chronic kidney disease or heart failure who should receive ACE inhibitors or ARBs [1]. Beta-blockers are generally not the first-line treatment for hypertension unless heart failure or myocardial infarction is present. Combination therapy should be considered if monotherapy fails, with evidence showing the reduction in blood pressure is about five times greater when drugs from two different classes are combined than when the dose of one drug is doubled. Combination therapy can be given as multiple pills or as a single-pill combination, and recent studies suggest that adherence and persistence rates are higher for single-pill combination therapy [58]. In Table 1 and below are summarized the primary indications and side effects of antihypertensive medications for the most common congenital heart diseases.

### 5.3 Inhibitors of renin–angiotensin–aldosterone system

ACEIs and ARBs have shown efficacy in hypertension and heart failure of any cause [59]. They are also recommended in hypertensive ACHD patients, particularly in those with systemic LV failure. ARBs should be considered to slow the progression of aortic dilatation in hypertensive ACHD with aortopathy [60]. Newer shreds of evidence recommend sacubitril/valsartan to substitute ACEI in ACHD patients who persist symptomatic regardless of optimal HF therapy [61].

Caveat for afterload reduction in Fontan patients: systemic venous return may pathologically fall and, in turn, also cardiac filling and output; for those with intra- or extra-cardiac defects, a right-to-left shunt may, on the other hand, rise and, thus, cause oxygen desaturation. Lastly, due to their potential adverse effects on the fetus should be discontinued during pregnancy.

This class of drugs exhibited a favorable effect on cognitive functions [62,63] and this should be taken into account in ACHD patients where the exposure to hypoxia, inflammation, and ECC may affect cognitive function [64].

### 5.4 Beta-blockers

Beta-blockers decrease heart frequency, contractility, and blood pressure, and, in turn, cardiac output and oxygen demand, promoting coronary vasodilation. First-generation drugs are non-selective beta-blockers targeting both beta 1 and beta 2 receptors (such as propranolol), second-generation ones are better cardio-selective [65] (such as atenolol), and third-generation blockers diversify selectivity for alfa1-receptors with vasodilatory peculiarities (such as nebivolol).

They should be the treatment of choice for hypertensive ACHD with heart failure. For example, bisoprolol has multiple beneficial mechanisms, as it not only blocks the b1-receptor but also produces endothelial nitric oxide, and decreases myocardial fibrosis and systemic vascular resistance [65]. Another example is propranolol, which should be considered for the treatment of hypertensive ACHD with heart failure caused by hypertrophic cardiomyopathy [66].

Furthermore, usage of beta-blockers may have a favorable function in hypertensive patients with a systemic RV, peculiarly at higher tolerated doses [65,66]. Lastly, in hypertensive ACHD with heart failure with preserved ejection fraction, beta-blockers may be instrumental by also ameliorating ventricular filling.

Caveat as beta-blockers may exacerbate conduction abnormalities, especially in congenitally corrected TGA. Also, they should be discontinued during pregnancy for their potential teratogenicity and/or detrimental effects on the conduction system in the fetus. Among the potential side effects of beta-blockers, as we are dealing with ACHD which is a younger population, particular considerations when prescribing this medication deserve the potential of erectile dysfunction.

### 5.5 Diuretics

Loop diuretics have not exhibited improved outcome of survival, but are commonly used in hypertensive ACHD, as successfully relieve symptoms, peripheral edema, and severity of dyspnoea, particularly in decompensated heart failure with preserved or reduced ejection fraction, even in Eisenmenger syndrome with a right-to-left shunt [1, 2, 67].

Potassium-sparing diuretics, especially spironolactone, improve the overall survival in ACHD patients with systemic hypertension and heart failure. Moreover, it improves cardiac and endothelial cell function and reduces inflammation in Fontan patients with protein-losing enteropathy.

Caveat as inappropriate dose increase of all diuretics can reduce preload in Fontan patients, dramatically decreasing cardiac output and/or causing the cardio-renal syndrome [67].

### 5.6 Calcium antagonists

This class includes dihydropyridine and non-dihydropyridine calcium channel blockers (CCBs). Dihydropyridines are powerful vasodilators, while non-dihydropyridines have an inferior capacity to vasodilate but strong negative chronotropic, dromotropic, and inotropic effects.

Dihydropyridines are a strategic component of combination therapies in hypertensive ACHD in whom more aggressive blood pressure control is needed. Nifedipine is efficacious and safe in pregnancy-induced hypertension [1,2,67,68]. Non-dihydropyridine calcium antagonists (either verapamil or diltiazem) are mainly used in hypertensive ACHD who also require rate control for the acute treatment and the long-term management of supraventricular arrhythmias (such as sino-atrial and atrio-ventricular node-dependent arrhythmias, multifocal atrial tachycardia) and ventricular tachyarrhythmias involving the Purkinje fibers (fascicular ventricular tachycardia).

Caveat as calcium antagonists (non-dihydropyridines) may have important negative inotropic and chronotropic effects in ACHD patients with systemic left ventricular failure, thus affecting ventricular performance and exercise capacity. Moreover, CCBs (non-dihydropyridines) can potentially unmask latent conduction system disease, especially in ACHD with pre-excitation or Ebstein anomaly of the tricuspid valve. Finally, in ACHD with shunt defects, dihydropyridines, such as all other vasodilators, may exaggerate the right-to-left shunt [1, 2, 67].

## 6 Chronic hypertension in pregnancy

Chronic hypertension is a complication in 1%–2% of pregnancies and is becoming more common, especially in women with congenital heart disease. Women with chronic hypertension have a higher risk of maternal and perinatal complications compared to those without hypertension.

Clinical guidelines define chronic hypertension as having a blood pressure of 140/90 mm Hg before pregnancy or before 20 weeks gestation [69,70]. Although no antihypertensive medication is a proven human teratogen, there have been associations between ACEIs, ARBs, beta-blockers, and birth defects, although these may have been influenced by residual confounding factors from underlying hypertension.

It is acceptable to continue using antihypertensive agents, including ACEIs, ARBs, and beta-blockers, until conception due to inconsistent contemporary literature [70], and, then, they should be discontinued after proven conception. This is particularly important for women taking ACEIs for renoprotection in chronic kidney disease (CKD) or beta-blockers for ventricular arrhythmias. As conception may take up to 12 months and women over 30 years are at greater risk for subfertility, replacing medication pre-pregnancy can mean suboptimal medication for 1–2 years [70].

Four national and international practice guidelines endorse “tight” blood pressure control for all forms of pregnancy hypertension, based on the results of the international Control of Hypertension In Pregnancy Study (CHIPS) [71,72]. The CHIPS trial achieved “tight” blood pressure control through an algorithm of antihypertensive up- or down-titration, using single or multiple medications. Therapy was decreased if diastolic blood pressure fell to 80 mm Hg or below and increased if systolic blood pressure was 160 mmHg, regardless of diastolic blood pressure for safety [71,72]. Other societies do not yet recognize the evidence to be conclusive. The American College of Obstetricians and Gynecologists (ACOG) recommends treating blood pressure emergently when it reaches severe levels (ie, 160/110 mm Hg) but not at all before then unless there are comorbidities pending [70].

The most commonly used and recommended antihypertensive medications in pregnancy come from different drug classes, including labetalol, nifedipine, hydralazine, methyldopa, and hydrochlorothiazide. All of these medications cross the placenta.

Labetalol is a combined alpha- and non-selective beta-blocker, as beta-blockade predominates the main effect is vasodilation without reduction of cardiac output or reflex tachycardia.

Nifedipine, as a dihydropyridine calcium channel blocker, reduces systemic vascular resistance by generating vasodilation.

Hydralazine is a direct-acting vasodilator that is commonly associated with reflex tachycardia, especially when employed as oral monotherapy; for this reason, it is used mainly intravenously.

Methyldopa is a centrally acting alpha-receptor antagonist that reduces peripheral vascular resistance by decreasing sympathetic tone.

Hydrochlorothiazide is supported as a second-line agent by ACOG, and ongoing use is not associated with volume depletion, while concerns about neonatal side effects are not supported by trials of thiazide use for preeclampsia prevention.

## 7 Conclusions

Systemic hypertension in patients with ACHD is commonly observed, and different mechanisms including the underlying cardiac disease, the development of metabolic syndrome and secondary HTN seem to play a role. Regular BP monitoring is mandatory for an early identification of HTN which, once confirmed, should be treated using different strategies, from lifestyle modifications to medical therapy.

The complex clinical management of different congenital physiopathology cannot and must not undermine or overshadow a successful treatment of systemic hypertension in adults with congenital heart disease.

## Funding

This research did not receive any specific grant from funding agencies in the public, commercial, or not-for-profit sectors.

## Declaration of competing interest

The authors declare that they have no known competing financial interests or personal relationships that could have appeared to influence the work reported in this paper.

## References

[1] Williams B., Mancia G., Spiering W. (2018 Sep 1). ESC scientific document group. 2018 ESC/ESH guidelines for the management of arterial hypertension. Eur Heart J.

[2] de Simone G., Mancusi C., Hanssen H. (2022 Sep 14). Hypertension in children and adolescents. Eur Heart J.

[3] Bauer U.M.M., Körten M.A., Diller G.P. (2019 Feb 15). Cardiovascular risk factors in adults with congenital heart defects - recognised but not treated? An analysis of the German National Register for Congenital Heart Defects. Int J Cardiol.

[4] Flannery L.D., Fahed A.C., DeFaria Yeh D. (2018 Feb 15). Frequency of guideline-based statin therapy in adults with congenital heart disease. Am J Cardiol.

[5] Roche S.L., Silversides C.K. (2013 Jul). Hypertension, obesity, and coronary artery disease in the survivors of congenital heart disease. Can J Cardiol.

[6] Gillett C., Wong A., Wilson D.G., Wolf A.R., Martin R.P., Kenny D. (2011 Feb). Underrecognition of elevated blood pressure readings in children after early repair of coarctation of the aorta. Pediatr Cardiol.

[7] Di Salvo G., Castaldi B., Baldini L. (2011 Dec). Masked hypertension in young patients after successful aortic coarctation repair: impact on left ventricular geometry and function. J Hum Hypertens.

[8] Canniffe C., Ou P., Walsh K., Bonnet D., Celermajer D. (2013 Sep 10). Hypertension after repair of aortic coarctation--a systematic review. Int J Cardiol.

[9] Vogt M., Kühn A., Baumgartner D. (2005 Jun 21). Impaired elastic properties of the ascending aorta in newborns before and early after successful coarctation repair: proof of a systemic vascular disease of the prestenotic arteries?. Circulation.

[10] Di Salvo G., Pacileo G., Limongelli G. (2007 Sep). Abnormal regional myocardial deformation properties and increased aortic stiffness in normotensive patients with aortic coarctation despite successful correction: an ABPM, standard echocardiography and strain rate imaging study. Clin Sci (Lond).

[11] Vriend J.W., de Groot E., de Waal T.T. (2006 Jan). Increased carotid and femoral intima-media thickness in patients after repair of aortic coarctation: influence of early repair. Am Heart J.

[12] Gidding S.S., Rocchini A.P., Moorehead C., Schork M.A., Rosenthal A. (1985 Mar). Increased forearm vascular reactivity in patients with hypertension after repair of coarctation. Circulation.

[13] Brili S., Dernellis J., Aggeli C. (1998 Nov 1). Aortic elastic properties in patients with repaired coarctation of aorta. Am J Cardiol.

[14] Brouwer R.M., Erasmus M.E., Ebels T., Eijgelaar A. (1994 Sep). Influence of age on survival, late hypertension, and recoarctation in elective aortic coarctation repair. Including long-term results after elective aortic coarctation repair with a follow-up from 25 to 44 years. J Thorac Cardiovasc Surg.

[15] Niwa K., Perloff J.K., Bhuta S.M. (2001 Jan 23). Structural abnormalities of great arterial walls in congenital heart disease: light and electron microscopic analyses. Circulation.

[16] Sehested J., Baandrup U., Mikkelsen E. (1982 Jun). Different reactivity and structure of the prestenotic and poststenotic aorta in human coarctation. Implications for baroreceptor function. Circulation.

[17] Ou P., Bonnet D., Auriacombe L. (2004 Oct). Late systemic hypertension and aortic arch geometry after successful repair of coarctation of the aorta. Eur Heart J.

[18] Ou P., Celermajer D.S., Mousseaux E. (2007 Feb 27). Vascular remodeling after "successful" repair of coarctation: impact of aortic arch geometry. J Am Coll Cardiol.

[19] Bassareo P.P., Marras A.R., Manai M.E., Mercuro G. (2009 May). The influence of different surgical approaches on arterial rigidity in children after aortic coarctation repair. Pediatr Cardiol.

[20] Morgan G.J., Lee K.J., Chaturvedi R., Bradley T.J., Mertens L., Benson L. (2013 Feb). Systemic blood pressure after stent management for arch coarctation implications for clinical care. JACC Cardiovasc Interv.

[21] Ladouceur M., Boutouyrie P., Boudjemline Y. (2013 Dec 24). Unknown complication of arterial switch operation: resistant hypertension induced by a strong aortic arch angulation. Circulation.

[22] Di Salvo G., Bulbul Z., Pergola V. (2017 Aug 15). Gothic aortic arch and cardiac mechanics in young patients after arterial switch operation for d-transposition of the great arteries. Int J Cardiol.

[23] Alpert B.S., Bain H.H., Balfe J.W., Kidd B.S., Olley P.M. (1979 Apr). Role of the renin-angiotensin-aldosterone system in hypertensive children with coarctation of the aorta. Am J Cardiol.

[24] Beekman R.H., Katz B.P., Moorehead-Steffens C., Rocchini A.P. (1983 Jul). Altered baroreceptor function in children with systolic hypertension after coarctation repair. Am J Cardiol.

[25] Polson J.W., McCallion N., Waki H. (2006 Jun 20). Evidence for cardiovascular autonomic dysfunction in neonates with coarctation of the aorta. Circulation.

[26] Ververs F.A., Eikendal A.L.M., Kofink D. (2022 Jul 19). Preclinical aortic atherosclerosis in adolescents with chronic disease. J Am Heart Assoc.

[27] Senzaki H., Iwamoto Y., Ishido H. (2008 Jan). Arterial haemodynamics in patients after repair of tetralogy of Fallot: influence on left ventricular after load and aortic dilatation. Heart.

[28] Williams J.C., Barratt-Boyes B.G., Lowe J.B. (1961 Dec). Supravalvular aortic stenosis. Circulation.

[29] Daniels S.R., Loggie J.M., Schwartz D.C., Strife J.L., Kaplan S. (1985 Feb). Systemic hypertension secondary to peripheral vascular anomalies in patients with Williams syndrome. J Pediatr.

[30] Longobardo L., Carerj S., Bitto A. (2021 Jun 22). Bicuspid aortic valve and aortopathy: novel prognostic predictors for the identification of high-risk patients. Eur Heart J Cardiovasc Imaging.

[31] Giordano R., Forno D., Lanfranco F., Manieri C., Ghizzoni L., Ghigo E. (2011 May). Metabolic and cardiovascular outcomes in a group of adult patients with Turner's syndrome under hormonal replacement therapy. Eur J Endocrinol.

[32] Nathwani N.C., Unwin R., Brook C.G., Hindmarsh P.C. (2000 Mar). Blood pressure and Turner syndrome. Clin Endocrinol.

[33] Cordina R.L., Celermajer D.S. (2010 Jun). Chronic cyanosis and vascular function: implications for patients with cyanotic congenital heart disease. Cardiol Young.

[34] Shankland S.J., Ly H., Thai K., Scholey J.W. (1994 Nov). Increased glomerular capillary pressure alters glomerular cytokine expression. Circ Res.

[35] Cooper T.J., Clutton-Brock T.H., Jones S.N., Tinker J., Treasure T. (1985 Jul). Factors relating to the development of hypertension after cardiopulmonary bypass. Br Heart J.

[36] Li D., Niu Z., Huang Q., Sheng W., Wang T. (2020 Aug 17). A meta-analysis of the incidence rate of postoperative acute kidney injury in patients with congenital heart disease. BMC Nephrol.

[37] Madsen N.L., Goldstein S.L., Frøslev T., Christiansen C.F., Olsen M. (2017 Sep). Cardiac surgery in patients with congenital heart disease is associated with acute kidney injury and the risk of chronic kidney disease. Kidney Int.

[38] Greenberg J.H., Zappitelli M., Devarajan P. (2016 Nov 1). TRIBE-AKI consortium. Kidney outcomes 5 Years after pediatric cardiac surgery: the TRIBE-AKI study. JAMA Pediatr.

[39] Kwiatkowski D.M., Price E., Axelrod D.M. (2017 Aug). Incidence, risk factors, and outcomes of acute kidney injury in adults undergoing surgery for congenital heart disease. Cardiol Young.

[40] Ohuchi H., Takasugi H., Ohashi H. (2004 Oct 26). Abnormalities of neurohormonal and cardiac autonomic nervous activities relate poorly to functional status in fontan patients. Circulation.

[41] Eindhoven J.A., van den Bosch A.E., Jansen P.R., Boersma E., Roos-Hesselink J.W. (2012 Nov 20). The usefulness of brain natriuretic peptide in complex congenital heart disease: a systematic review. J Am Coll Cardiol.

[42] Ross R.D., Daniels S.R., Schwartz D.C., Hannon D.W., Shukla R., Kaplan S. (1987 Apr 1). Plasma norepinephrine levels in infants and children with congestive heart failure. Am J Cardiol.

[43] Morgan C., Al-Aklabi M., Garcia Guerra G. (2015 Aug 11). Chronic kidney disease in congenital heart disease patients: a narrative review of evidence. Can J Kidney Health Dis.

[44] Takken T., Giardini A., Reybrouck T. (2012). Recommendations for physical activity, recreation sport, and exercise training in paediatric patients with congenital heart disease: a report from the exercise, basic & translational research section of the European association of cardiovascular prevention and rehabilitation, the European congenital heart and lung exercise group, and the association for European paediatric cardiology. Eur J Prev Cardiol.

[45] Moola F., Fusco C., Kirsh J.A. (2011). The perceptions of caregivers toward physical activity and health in youth with congenital heart disease. Qual Health Res.

[46] Cheuk D.K., Wong S.M., Choi Y.P., Chau A.K., Cheung Y.F. (2004 Apr). Parents' understanding of their child's congenital heart disease. Heart.

[47] Caterini J.E., Campisi E.S., Cifra B. (2020 Sep). Physical activity promotion in pediatric congenital heart disease: are we running late?. Can J Cardiol.

[48] Jepson A., Danford D., Cramer J.W., Tsai S., Yetman A.T. (2022 Oct). Assessment of hypertension, guideline-directed counseling, and outcomes in the ACHD population. Pediatr Cardiol.

[49] Afilalo J., Therrien J., Pilote L., Ionescu-Ittu R., Martucci G., Marelli A.J. (2011 Sep 27). Geriatric congenital heart disease: burden of disease and predictors of mortality. J Am Coll Cardiol.

[50] Moons P., Van Deyk K., Dedroog D., Troost E., Budts W. (2006 Aug). Prevalence of cardiovascular risk factors in adults with congenital heart disease. Eur J Cardiovasc Prev Rehabil.

[51] Goldstein S.A., D'Ottavio A., Spears T. (2020 Jul 21). Causes of death and cardiovascular comorbidities in adults with congenital heart disease. J Am Heart Assoc.

[52] Verheugt C.L., Uiterwaal C.S., van der Velde E.T. (2010 May). Mortality in adult congenital heart disease. Eur Heart J.

[53] Labombarda F., Hamilton R., Shohoudi A. (2017 Aug 15). AARCC. Increasing prevalence of atrial fibrillation and permanent atrial arrhythmias in congenital heart disease. J Am Coll Cardiol.

[54] Tomek J., Bub G. (2017 Jun 15). Hypertension-induced remodelling: on the interactions of cardiac risk factors. J Physiol.

[55] Verdecchia P., Schillaci G., Guerrieri M. (1990). Circadian blood pressure changes and left ventricular hypertrophy in essential hypertension. Circulation.

[56] Hoshino A., Nakamura T., Matsubara H. (2010). The bedtime administration ameliorates blood pressure variability and reduces urinary albumin excretion in amlodipine-olmesartan combination therapy. Clin Exp Hypertens.

[57] Ekelund U., Luan J., Sherar L.B., Esliger D.W., Griew P., Cooper A. (2012 Feb 15). International Children's Accelerometry Database (ICAD) Collaborators. Moderate to vigorous physical activity and sedentary time and cardiometabolic risk factors in children and adolescents. JAMA.

[58] Tsioufis K., Kreutz R., Sykara G., van Vugt J., Hassan T. (2020 Jun). Impact of single-pill combination therapy on adherence, blood pressure control, and clinical outcomes: a rapid evidence assessment of recent literature. J Hypertens.

[59] Goodfriend T.L. (2000). Angiotensin receptors: history and mysteries. Am J Hypertens.

[60] Shen Y.H., LeMaire S.A. (2017). Molecular pathogenesis of genetic and sporadic aortic aneurysms and dissections. Curr Probl Surg.

[61] Fusco F., Scognamiglio G., Merola A. (2022 Dec 2). Safety and efficacy of sacubitril/valsartan in patients with a failing systemic right ventricle: a prospective single-center study. Circ Heart Fail.

[62] Deng Z., Jiang J., Wang J. (2022 Oct). Alzheimer's disease neuroimaging initiative†. Angiotensin receptor blockers are associated with a lower risk of progression from mild cognitive impairment to dementia. Hypertension.

[63] Tedesco M.A., Ratti G., Di Salvo G., Natale F. (2002). Does the angiotensin II receptor antagonist losartan improve cognitive function?. Drugs Aging.

[64] Naef N., Ciernik A., Latal B., Liamlahi R. (2023 Jan 7). Children's Heart and Development Research Group. Hippocampal volume and cognitive performance in children with congenital heart disease. Pediatr Res.

[65] Dézsi C.A., Szentes V. (2017). The real role of blockers in daily cardiovascular therapy. Am J Cardiovasc Drugs.

[66] Ramakrishnan S., Ghati N., Ahuja R. (2021). Efficacy and safety of propranolol in infants with heart failure due to moderate-to-large ventricular septal defect (VSD-PHF study)—a prospective randomized trial. Ann Pediatr Cardiol.

[67] Brida M., Diller G.P., Nashat H. (2019 Apr 1). Pharmacological therapy in adult congenital heart disease: growing need, yet limited evidence. Eur Heart J.

[68] Bellos I., Pergialiotis V., Papapanagiotou A., Loutradis D., Daskalakis G. (2020 Oct). Comparative efficacy and safety of oral antihypertensive agents in pregnant women with chronic hypertension: a network metaanalysis. Am J Obstet Gynecol.

[69] Scott G., Gillon T.E., Pels A., von Dadelszen P., Magee L.A. (2022). Guidelines—similarities and dissimilarities: a systematic review of international clinical practice guidelines for pregnancy hypertension. Am J Obstet Gynecol.

[70] Magee L.A., Khalil A., Kametas N., von Dadelszen P. (2022). Toward personalized management of chronic hypertension in pregnancy. Am J Obstet Gynecol.

[71] Magee L.A., von Dadelszen P., Singer J. (2016). The CHIPS randomized controlled trial (Control of Hypertension in Pregnancy Study): is severe hypertension just an elevated blood pressure?. Hypertension.

[72] Magee L.A., von Dadelszen P., Rey E. (2015). Less-tight versus tight control of hypertension in pregnancy. N Engl J Med.

